# A Qualitative Study on How Entrustable Professional Activities Support Medical Students in Their Transitions across Clerkships

**DOI:** 10.5334/pme.825

**Published:** 2023-05-29

**Authors:** Anne E. Bremer, Larissa I. A. Ruczynski, Petra Bot, Cornelia R. M. G. Fluit, Marjolein H. J. van de Pol

**Affiliations:** 1 Research on Learning and Education, Radboudumc Health Academy, Radboud University Medical Center, Nijmegen, The Netherlands; 2 Radboud Amalia’s Children Hospital, Radboud University Medical Center, Nijmegen, The Netherlands.; 3 Department of Radboudumc Health Academy, Radboud University Medical Center, Nijmegen, The Netherlands; 4 Department of Primary and Community Care, Radboud University Medical Center, Nijmegen, The Netherlands

## Abstract

**Introduction:**

Medical students regularly transition between clerkships. These transitions can lead to discontinuity in their development because of the need to adapt to a new environment. The use of entrustable professional activities (EPAs) might facilitate less disruptive transitions across clerkships, as they could provide support at the start of a clerkship. This study aims to shed light on how an EPA-based curriculum contributes to medical students’ learning processes during transitions.

**Methods:**

The authors used a constructivist rapid ethnographic design. They conducted observations and interviews with 11 medical students in their Pediatrics clerkship; six of them were in clerkships not utilizing EPAs, and five were using EPAs. Data collection was followed by template analysis such that all data were coded with a template that was continually updated until the authors all agreed upon a definitive template.

**Results:**

Four themes proved important when considering the impact of EPAs during transitions between clerkships: transitions as a learning opportunity, building relationships in context, taking leadership in the landscape of practice and feedback-seeking behavior.

**Discussion:**

EPAs smooth clerkship transitions, as they establish continuity in the student’s development and facilitate navigating discontinuity in transitions. Students build skills and confidence in order to grow and work with increasing independence within the clerkships. Transitions offer important learning opportunities for students, which can be fully exploited by using EPA guidance.

## Introduction

Transitions are everyday business in medical education. The most important transitions are those from preclinical to clinical education [1], from medical student to physician [2], and from physician to medical specialist [3,4]. Medical students regularly transition from one workplace to another during their clerkships [5], where they have to adapt to a new environment and its practices and norms. Clerkships provide excellent learning opportunities as medical students primarily develop their knowledge and skills through clinical work [6], and early clinical work offers students the opportunity to convert knowledge into clinical competence [7]. Particularly boundaries, or transitions, across different clerkships create great space for learning [8].

Transitions offer a great opportunity for learning and growth [9], but they also require a “fundamental re-examination of who and what we are, even if this processing is occurring at a largely unconscious level” [10]. Due to habituation problems [11], however, transitions can also lead to discontinuity in the students’ professional development [11,12]. This might give rise to negative emotions such as anxiety or sadness, which are likely to be related to the intensifying responsibilities that medical students experience with each transition [13,14,15], as they move towards more independent practice [16]. Little is known about how to facilitate transitions for medical students in their clerkships.

To resolve this discontinuity when transitioning between clerkships, students have to develop and change their behavior to integrate into the new environment [12]. Though discontinuity may give rise to feelings of stress and discomfort, such problems can be reduced if students are prepared for the transition [17]. Yardley and colleagues [18] explain the importance of stimulating appropriate learning in practice and building independence while being actively supported and mentored. Useful feedback, increasing responsibility and trust and support from a supervisor can encourage this, and might be be supported by using entrustable professional activities (EPAs). EPAs are “units of professional practice that can be fully entrusted to a trainee, as soon as he or she has demonstrated the necessary competence to execute this activity unsupervised” [19]. With the use of EPAs, students gradually gain trust from their supervisors to perform clinical activities by themselves, and they thus become increasingly independent and responsible. This could mitigate the negative aspects of navigating discontinuity and facilitate better transitions across different clerkships.

Different clerkships taken together can be considered as *landscapes of practice (LOP)* [20] which relate to communities of practice (COP) [21]. In COPs, learners develop their professional identity when moving towards full participation in the workplace [22]. People in this workplace, or a community of practice, form a group with a shared passion or concern [21]. In contrast to COPs, LOPs take into consideration not only moving to full participation in a single workplace, but also moving between communities [23]. Moving in a landscape with different practices enables learners to develop a professional identity, even more so than in one community of practice [24]. Clinical clerkships comprise both LOPs and COPs, as medical students move both within and between communities.

Research shows that feelings of stress or nervousness hindered medical students’ learning processes, but the context has great power to optimize the learning opportunities of medical students [8]. This study also shows great opportunities for learning in boundary crossings, e.g., when “a student and a supervisor visited another ward and different perspectives intersected and new possibilities for learning arose” [8]. Thus, knowing how to cross borders is vital for medical students’ professional development as it provides great learning opportunities. It is crucial, therefore, to build curricula in which medical students are supported in transitioning and crossing borders to multiple landscapes of practice by feedback from their supervisors.

The Radboud University Medical Center in Nijmegen, the Netherlands, introduced EPAs into their undergraduate curriculum in 2019. With the use of EPAs, students gradually gain trust from their supervisors to perform clinical activities by themselves, and they thus become increasingly independent and responsible. The implementation of an EPA-based curriculum means that medical students are required to prepare a personal training plan including personal learning goals, and to request feedback on a daily basis, both as retrospective entrustment-supervision scale grades and as narrative retrospective feedback. Students are expected to gather 25 feedback reports in a clerkship that lasts for 4 weeks (and 50 in clerkships lasting 8 weeks etc.). All feedback reports are collected in a personal e-portfolio, which provides an overview of their EPA development and their professional development. Working with EPAs, therefore, stimulates medical students to regularly request and receive feedback, which then quickly turns into a habit.

The received feedback should evidently be of good quality for it to be useful. According to Voelkel et al. [25], feedback should (1) be detailed and clear, (2) say exactly how to improve, (3) be constructive and authentic, (4) include positive aspects of work, and (5) justify the received mark. Research of Molloy et al. [26] and Pelgrim et al. [27] explains that feedback is typically considered as an act of teachers, by both students and teachers themselves. Though, in their framework they show that students actually are key agents in the process of seeking feedback, and that they can become engaged utilizers of the feedback they receive. This might be stimulated by EPAs, as they encourage students in their feedback seeking behavior [28].

Halfway through the clerkship, students in our new curriculum discuss their development with their supervisor, just as at the end of the clerkship for the final evaluation. As the curriculum is built on a block rotation structure, involving regular transitions to other clinical rotations, on the other hand, students may frequently experience discontinuity in their learning environment as soon as they rotate to different COPs. This forces students to take new positions all over again, which could hinder their learning. All this emphasizes the importance of facilitating smooth transitions for medical students.

We hypothesize that the use of EPA portfolios with frequent feedback moments facilitates less disruptive transitions across the different COPs in clerkships. It could mitigate the negative aspects of navigating discontinuity, and it could be the sliding scale [18] to decreasing supervision, as medical students become increasingly trusted by their supervisors. However, little is as yet known about how medical students can experience less stress and fewer insecurities at the start of a clerkship [28], and how an EPA-based curriculum may impact on this. In this study, we aim to shed light on how an EPA-based curriculum contributes to learning processes of medical students in transitions.

## Methods

In this study, we used a constructivist rapid ethnographic approach. Constructivism implies a reciprocal relationship between the participant and the researcher, with the researcher taking a reflexive stance [29] and trying to understand the data in the context of the whole [30]. Rapid ethnography is a research method which includes a narrowed research focus and important key participants in order to obtain rich research data [31]. With rapid ethnography, fieldwork takes place in a short and explicit period of time, in contrast to more traditional ethnographic research which usually takes months or years [32], which makes it very suitable for medical education research.

The research group consisted of educational scientists and medical doctors from different specialties, which allowed them to view the data from different perspectives. The lead author (Anne Bremer, AB) is an educationalist; Larissa Ruczynski (LR) is a medical doctor and researcher; Cornelia Fluit (CF) is a professor in workplace learning with a medical and educationalist background; Marjolein van de Pol (MP) is an associate professor of student wellbeing, general practitioner and educational researcher; and Petra Bot (PB) is a pediatrician and clinical supervisor.

### Setting

This study took place in the undergraduate curriculum of the Radboud University Medical Center in Nijmegen, the Netherlands. The undergraduate curriculum consists of two phases: a three-year Bachelor’s program and a three-year Master’s program. In the Master’s curriculum, students alternate between short periods of in-class teaching and longer periods of practice (clerkships), lasting 4 to 8 weeks. In the clerkships, students use an e-portfolio with five different EPAs (the EPA portfolio): (1) medical consultation, (2) medical procedures, (3) guidance and education, (4) communication and collaboration and (5) non-clinical activities, with students requesting daily feedback from their supervisors (both narrative and retrospective entrustment-supervision scale grades).

In this study, we conducted interviews and performed observations with two different groups of students: (1) medical students from the last cohort of the “old” curriculum (not working with EPAs), and (2) medical students from the first cohort of the “new” curriculum (working with EPAs). Besides this, there are no differences between the curricula. See Table 1 for an overview of the feedback and assessment processes of both curricula.

**Table 1:** Feedback and assessment processes of medical curricula with- and without EPAs.


	SIMILARITIES BETWEEN THE CURRICULA	DIFFERENCES BETWEEN THE CURRICULA

**Order and content of the clerkship**	All students attend block oriented clerkships in the same order (e.g. they all start with Internal Medicine, followed by Neurology/Psychiatry etc.), for 4–8 weeks each.	–

**Feedback**	During the clerkship, the students collect feedback from their supervisors.	Students who work **without** EPAs, irregularly collect feedback (often once or twice a week), and they write it down in their ‘feedback notebook’. Students who work **with** EPAs, regularly collect feedback (once or twice a day) and write it down in a personal e-portfolio. The supervisor validates the feedback.

**Assessment**	At the end of a clerkship, a supervisor evaluates the student based on the collected feedback and their own perception.	Students who work **without** EPAs are evaluated by the self-described feedback in the notebook combined with an assessment interview, to decide readiness for the next clerkship. Students who work **with** EPAs, are evaluated by the feedback in the e-portfolio (which is more extensive and detailed than the notebook’s feedback), combined with an evaluation interview to decide readiness for the next clerkship. The feedback is provided by more supervisors and validated by the feedback provider.


**Figure 1:**
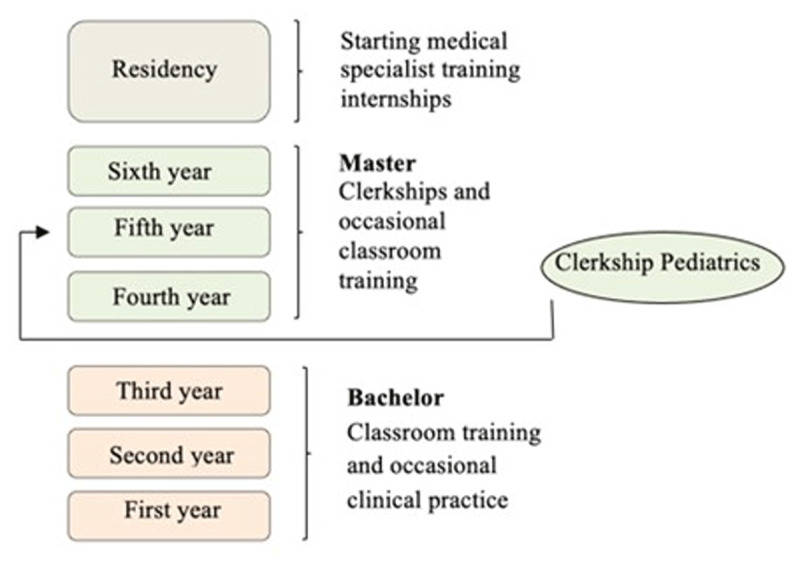
The medical curriculum of The Netherlands.

At the time of observation, all participants were second year students in the first week of their pediatrics clerkship, which has a duration of four weeks, For more information about the structure of the Dutch medical curriculum, see Figure 1. We chose participants from these groups because of their familiarity with practice, transitions and the EPA portfolio (the new curriculum students). See Appendix 1 for an overview of the Master’s curriculum with the different clerkships (episodes).

### Participants

We used a purposive sampling method to select the participants. AB met the students from both groups, who were about to embark on their pediatrics clerkship, in a regular educational meeting. The students were informed about the research and invited to participate. Six out of eight of the old curriculum students (OCS) volunteered, and five out of seven of the new curriculum students (NCS), which makes a total number of eleven participants. We used a qualitative design with multiple methods: observations and interviews.

### Observations

Prior to the observations, we agreed with the pediatrics staff and the participants on suitable moments for observations. The participants were unaware of the specific research question, but knew that the research focused on EPAs. Suitable moments were defined as moments with interaction between the participant and others (peers, supervisors, other staff). Different potential feedback moments were observed: briefings, educational sessions, feedback moments and preparation of clinical consultations. AB, who is not a medical doctor, carried out the observations over two weeks, with an open mind and without being biased by the daily affairs of a clinical setting. She was trained to undertake observations and the observations were supervised by an experienced observational researcher.

### Interviews

AB conducted the interviews, which lasted between 40–60 minutes. AB is a trained interviewer with previous experience in interview studies. We used a semi-structured interview guide that was constructed by AB, CF and MP. The guide was mostly based on the following synthesizing concepts: transition, feedback, landscapes of practice and EPAs (the last one only in the NCS group). The interview guide contained fixed questions based on the synthesizing concepts, which were presented to every participant. The above-mentioned fieldnotes and drawings resulting from the observations provided insight into participant specific situations, which allowed us to iteratively add questions to the interview guide, only presented to that specific participant. Furthermore, participants were actively encouraged to introduce new subjects themselves. The interview took place as soon as possible after the observation period.

We conducted 11 interviews and 35 hours of observations of six OCS (3 males, 3 females) and five NCS (2 males, 3 females) between November 2019 and February 2020. By observing a student in her or his first week of a new clerkship, the researcher gained insight into how it feels to be thrown into the deep end when entering a new environment.

### Data analysis

The observations were not audio- or video recorded, but handwritten fieldnotes of dialogues, roles and activities were made, as well as drawings of specific space settings. All field data were anonymized after the interviews took place. The fieldnotes and drawings of the observations were promptly transformed into descriptive reports by AB, and she transcribed the interviews verbatim within two weeks. We used a template analysis (TA) to analyze the data, as proposed by the template analysis model by Brooks et al. [33]. Template analysis is a relatively recent development in qualitative healthcare research [34] and very useful for investigating the viewpoints of different groups in an organizational setting [35]. Because of the flexible coding structure, TA “allows researchers to explore the richest aspects of data in real depth” [33]. Guided by TA, we applied the following strategy:

1. We (AB, CF, MP and LR) created the following a priori defined codes based on literature: “transitions within the LOP”, “continuity in learning”, “feedback”, “EPAs”, and “impact of the context”.
2. We read the interviews and the observation reports to familiarize ourselves with the data.
3. We established an initial template with preliminary themes based on the a priori defined themes, and new themes were added to the template. AB and LR then coded all interviews with this first template, followed by a discussion with the whole research group about the explored data and the template. All disagreements were thoroughly discussed until general consensus was reached.
4. We adjusted the template, and this was followed by a next round of coding by AB, LR, CF and MP.
5. We discussed the themes and relevant data and we formulated new (sub)themes. Eventually, a definitive template was agreed upon, with triangulation, by all the researchers involved. This template was applied to all interview and observation data in order to conduct an exhaustive concrete analysis. Atlas.ti was used to process the data.

### Ethical approval

The ethical approval has been granted for studies involving human subjects by the ethics committee of the Netherlands Association of Medical Education (NVMO), reference number 2019.7.8.

## Results

We identified that the EPA-based curriculum solicited different behaviors in students when compared to those that were not exposed to EPAs. Based on our analysis, we defined four themes related to learning during clerkships where EPAs can make a difference: “transitions as a learning opportunity”, “building relationships in context”, “taking leadership in the landscape of practice” and “feedback-seeking behavior”. A prevailing view amongst all NCS with regard to the transition across clerkships was that EPAs provide guidance at the start of a clerkship and promote the search for new learning experiences in the workplace:

“Well, I think the EPAs do give you a kind of grip on things as you just have to attain several practice assessments and you do need to complete these EPAs at a certain level. So they force you to mix with the team and show them, hey, I’m here, and I want some feedback, because I’ve done this. So they do force you to show them that you’re actually there and that you need to attain the required practice assessments.” (NCS)

There were also several similarities between the two groups. All participants described the uncertainties that come with starting a new clerkship, and the effect of the medical department and its staff on the transition: “Always slightly nerve-racking. Each clerkship is different, the doctors are different, and you need to adjust to their personalities. In surgery, for instance, I was wondering how far I should go with my questions, or what exactly I was expected to do. It really took some adjusting.” (OCS)

The most striking findings of the above-mentioned four themes are presented in Table 2, and the themes are discussed in detail below, moving from a broader to a more specific context.

**Table 2:** Key findings related to learning during clerkships with and without EPAs.


THEME	KEY FINDINGS	KEY FINDINGS RELATED TO EPAS

(1) Transitions as a learning opportunity	- Transitions are difficult due to the pressure to perform well, but experiences gained in previous clerkships benefit the adaptation to a new clerkship.	- EPAs offer NCS guidance at the start of their new clerkship. - The use of the e-portfolio increases the student’s confidence and lowers stress levels.

(2) Building relationships in context	- Being part of the team supports transitioning and learning in clerkships. - A mentor could help students in their enthusiasm and make them being prepared to do more.	

(3) Taking leadership in the landscape of practice	- Regular feedback moments motivate self-reflection.	- EPAs support taking leadership over the student’s own actions and responsibilities, which is conducive to learning. - Working with EPAs encourages students to be assertive and to show initiative, which supports learning.

(4) Feedback-seeking behavior	- Regular feedback moments stimulate learning and transitioning across clerkships. - Without the stimulation of regularly asking for (immediate) feedback (e.g. without using EPAs) results in less and short feedback moments. - Asking for feedback evidently creates ambiguous feelings for students as they are scared to ‘bother’ their supervisor.	- EPAs stimulate feedback-seeking behavior by the need to ask for feedback on a regular basis to write down in the e-portfolio. - EPAs support students and supervisors to make more time for feedback moments.


### Transitions as a learning opportunity

Students in both curricula agreed that embarking on a new clerkship did not mean starting all over again, but it did launch a period of adjusting and getting used to new circumstances, which required much effort. However, certain conditions were identified that may have helped to smooth the transition. OCS indicated that the provision of clear and extensive information provided by supervisors at the start of a new clerkship helped them to get going. Besides this, previous clinical experience with patients made transitions easier and supported their move into a new workplace. Students always bring previous experiences with them, which benefits their adaptation to a new clerkship.

EPAs appeared to offer NCS guidance at the start of their new clerkship. They were not always explicitly aware that it were the EPAs that supported them in their transition, but repeated performance of certain activities strengthened their confidence and guided them in transitions. In this way, EPAs indirectly support transitions, as they encourage the repeated performance of activities in clerkships: “Yes, you do develop your skills, of course, and I think it’s more about what you’ve done than about what you’ve recorded in your file. At the end of the day, it’s about developing your skills, whether you write them down or not.” (NCS)

Transitions are more difficult when a clerkship, or a community of practice, differs greatly from the previous one. A more similar landscape of practice facilitates a smoother transition, partly because of the confidence students gained in their previous clerkship. Confidence, however, is based not only on previous experience but also on the responsibilities acquired and the activities undertaken. For NCS, these activities were captured in their personal e-portfolios. NCS indicated that recognizing their own capabilities and viewing them in their e-portfolios increased their confidence and lowered stress levels about their own capabilities.

Participants of both groups pointed out that transitions across clerkships were always difficult because of the pressure to perform well. The more experience, the easier it gets, but pressure to perform well will always be part of being an intern.

### Building relationships in context

OCS indicated that more direct supervision might support them in learning and transitioning to a new environment. Being observed by a supervisor and receiving feedback afterwards guides the learning process and supports the transition. Greater continuity in supervision might also support their learning process. This was indicated in one of our students’ responses to the idea of being supervised by the same supervisor for a longer period of time:

“I think it will have a great deal of influence because I think that you can get more out of it. Suppose they just need to watch you just once, which might be unfeasible, but let’s suppose that would be possible. Then I think they want to see you once, and that you start building trust early on, and then you have a foundation that would allow you to do more next time. So you have already put the foundation in place instead of having to prove yourself over and over again with every new doctor.” (OCS)

Maintaining a good relationship with the supervisor and a sense of belonging in the team fosters a smooth transition. NCS and OCS mentioned that being part of the team supported transitioning and learning. The specific workplace conditions and its degree of focus on education can support or hinder learning and transitions. Colleagues and the workplace account for an important part of transitioning and workplace learning success:

“…if you had a kind of mentor − perhaps that’s a personal thing for me − somebody who’s your supervisor and who you know. Someone like that could inspire more enthusiasm. I’ve got the feeling that if you’re just left to your own devices, and if it’s all down to you, well, you know, some can do that and some can’t. My feeling is that a designated contact would help. And then, once your enthusiasm has been boosted, I think you can get more out of it as you’ll be prepared to do more.” (NCS)

Even though this specific NCS indicates that a ‘kind of mentor’ could boost their motivation, NCS in general are more in contact with their supervisors because of the need to ask for feedback. This is EPA curriculum specific and it increases the student’s feedback-seeking behavior.

### Taking leadership in the landscape of practice

According to our participants, taking leadership over your own actions and responsibilities is conducive to learning, which is supported by the use of EPAs, as they stimulated our students to take the initiative in activities and responsibilities appropriate to their level of skills and experience. Two NCS explained how they took initiative on the first day of their new clerkship. For example:

“Take some initiative. If you have nothing to do, just watch what other people are doing, or tell them: I’ve got nothing to do, so if I can help you, let me know. And perhaps they give you silly chores like phone calls or dismissal letters or things like that. But it’s been my experience that if you take initiative, you realize more what needs to be done and you get more responsibilities. And it improves your popularity in the workplace.” (NCS)

Adopting a proactive attitude supports the learning process, but for OCS this was not always easy. These students showed less initiative in creating learning opportunities than NCS. NCS explained that working with feedback reports and EPAs encouraged them to be assertive and to show initiative, also when they had just started work in a new clerkship.

### Feedback-seeking behavior

Participants in both groups clearly indicated that it is of great importance for learning and transitioning across clerkships to receive sufficient feedback. Students in the new curriculum explained that, with the use of EPAs, requesting and receiving feedback was greatly stimulated, resulting in many opportunities for feedback and reflection. EPAs made it easier for students to ask for feedback:

“Erm, well I think that in the old curriculum they simply get less [feedback] because I know that people say that they have this thing completed in their first three or four clerkships and then they let it slip. So I guess that in that case you simply stop asking for feedback at some point. But we are actually actively stimulated to ask for feedback, which is a good thing, I think, because each time you get a reminder, you know, to improve this or that. So I think it does really have an effect if you’re actively involved and really take your practice assessments seriously.” (NCS)

The feedback they received, moreover, also motivated them to reflect critically on themselves: “So every time when I think I can attain a particular level but someone gives me lower scores, I will always go in search of what I should have done differently in order to do better next time. So it is a genuine incentive, so to speak, to keep developing.” (NCS)

OCS, furthermore, pointed out that feedback immediately after an activity takes place is of great importance. This happened infrequently for these students, as they usually only received feedback a few times at the end of the day in the course of a clerkship, when they requested feedback from their supervisors for their evaluation notebook.

During the observations, we noticed two different situations in feedback-seeking behavior. In the first situation, students asked for supervisor feedback in the hallway after seeing a patient. This often resulted in a very short, oral feedback moment while walking through the hallway, with the supervisor summing up a few feedback elements without providing written feedback. This situation happened often with OCS. In the second situation, feedback was requested after a patient had walked out the door when the supervisor and the student were still in the consultation room. This situation left more room for a longer feedback moment and for it to be written down. This happened often with the NCS, as they insisted on gathering feedback on their EPAs for their e-portfolios.

OCS explained that time for appropriate direct feedback was often lacking: “I think they just don’t have enough time to sit down and listen to you. They also tended to assume you already mastered things, that it was simply self-evident for you to do certain things.” (OCS)

Furthermore, they feel bad about taking valuable time from their supervisors: “Well, in my previous clerkship that was something of a lesson for me, you know, because in my role as an intern I always felt a little diffident to be taking up a doctor’s time. I guess it’s only worse for the new interns ‘cause they have even more feedback points.” (OCS)

This quote shows that OCS were reluctant to ask for even more feedback. In practice, students in the new curriculum also sometimes experienced ambiguous feelings when they frequently needed to ask for feedback and were afraid of disturbing the relationship with their supervisors:

“Thing is, well, yes, I do think that if you’re obliged to ask for a hell of a lot of feedback, as we are required to do, this may have a negative effect on your relations with your supervisor. I don’t normally feel a lot of stress about content stuff or theory or that sort of thing. But I do worry about, well, I’ve done something, and this doctor is super busy, and on top of that I need to bother him for feedback. It’s an additional burden that’s causing me quite a lot of stress.” (NCS)

On the other hand, EPAs gave NCS the advantage of creating opportunities for immediate feedback, as feedback reports are usually completed immediately after the activity occurred. Such feedback is appreciated: “That was great because he told me straightaway what I did right, and if I wanted to I could look into this or that and come back later. So I got my feedback, I’ve done well, no need to worry. Erm and I should check on a couple of things. So I liked that a lot.” (NCS)

The NCS agreed that frequently asking for feedback on EPAs can cause ambiguous feelings, but because of the resulting confidence they felt about their own abilities it is also a support for learning in the workplace. The EPAs guided them in finding motivation for self-development, and they stimulated them to actively search for points of improvement. Students also indicated, however, that it was generally empowering for them to build up their own skills, and not only because they were using EPAs.

Students in both curricula indicated that kindness of supervisors and specific feedback were “the key to learning”. OCS indicated that they often struggled when they received ambiguous feedback, which was counterproductive and left them feeling insecure. Besides this, OCS indicated that they often needed to memorize the feedback they received in their minds without recording it, whereas the NCS wrote it down in their e-portfolios.

## Discussion

Our findings show how students navigate transitions across clerkships and utilize learning opportunities, both with and without the guidance of an EPA-based curriculum. Our most important and novel finding is that EPAs support medical students in their transitions across clerkships. By providing guidance, stimulating feedback-seeking behavior on a broad array of EPAs, and encouraging initiative in the medical department, they mitigate the negative aspects of navigating discontinuity. Furthermore, EPAs reduce the perceived tension at the start of a new clerkship.

### Navigating discontinuity in learning with the use of EPAs

Transitions across clerkships may cause discontinuity in the students’ development [11,12] because it takes a change of behavior and professional development to get used to a new workplace [12]. Students should be well prepared for transitions in order to navigate the discontinuity associated with transitions and to be able to develop as medical professionals [17]. This study shows that EPAs, by stimulating feedback-seeking behavior, support students in establishing continuity in their learning process, as they allow them to look back at what they experienced and learned in previous clerkships. In this way, students become important utilizers of feedback information [26], and they carry this knowledge into the next clerkship. Our results indicate that feelings of uncertainty (experienced by both OCS and NCS), which can impede learning processes, can possibly be diminished if students deliberately review their previously received feedback and feel strengthened by doing so. NCS use EPAs as a reinforcing tool for feeling more comfortable, but OCS lack this kind of instrument.

The timing of feedback is important for continuity in learning. An EPA-based curriculum requires regular feedback moments that should generally take place immediately after the activity [36]. This creates an important moment for both NCS and supervisors, often resulting in valuable conversations with oral and written feedback. OCS, however, who do not receive feedback regularly, do not often have such conversations. This lack of regular feedback moments, which are so instructive for students, could have a negative impact on their learning process [28]. Working with EPAs, therefore, creates continuity in the feedback process and stimulates learning.

EPAs play an important role in inspiring students to engage in active self-development and self-improvement. When a transition evokes feelings of fear or insecurity [14], EPAs support NCS in focusing on their learning objectives and taking action in order to develop. OCS also focus on their learning objectives but receive less guidance and take less initiative in their curriculum without EPAs. Even if NCS are not always aware of the EPA’s motivational aspects, they do build up skills by repeated practice initiated by the EPA-based curriculum, which builds their confidence for transitioning across clerkships.

### Transitioning within the landscape of practice supported by EPAs

Transitioning across clerkships is likely to be difficult and comes with many insecurities about one’s own personality, skills and knowledge [10]. EPAs do not eliminate these insecurities for NCS, as pressure to perform will always exist. They do, however, build confidence and diminish stress because they give students the possibility to review their feedback and experiences in the e-portfolio. Whereas OCS tended to focus more on the context and the comfort provided by its people to reduce stresses, NCS used this as well, but in addition they also used their e-portfolios to lower stress levels.

Even though feelings of stress during transitions can hinder learning, transitions also offer a great space for learning [8,9]. This emphasizes the importance of looking beyond the difficulties involved in transitions towards the learning opportunities they offer. The context plays a considerable role in optimizing these learning opportunities within a LOP [8]. Our participants confirm this: building good relationships with colleagues and supervisors, and being part of the team fosters learning and growth at the boundaries of the COPs. EPAs, moreover, providing useful feedback and supervisor trust and support which are essential for acquiring independence, also appear to be meaningful in progressively implementing independence practice in transitioning across different COPs. A focus on increasing the learning opportunities inherent in transitions, therefore, will help to improve students’ development.

### Strengths and limitations

Firstly, we compared two groups that were enrolled in the same clerkship routine, but with different feedback systems. This provided insights into the two different curricula, and highlighted differences and similarities, which we can use for the further development of the Master’s program. Secondly, we used the unfamiliarity of AB with the context of a hospital setting as an advantage, as this gave us the opportunity to examine the medical department in a way similar to that of a medical student embarking on his or her first day of a new clerkship.

We acknowledge that this study also has limitations. Firstly, it was conducted in one university context only. Further research could examine the role of EPAs in clinical learning environments across different university programs in different contexts. Secondly, in order to study medical students during their transitions with greater thoroughness, further research could include observing students for two or more consecutive clerkships. Thirdly, we only included students in clerkships in a hospital setting, and to examine how they experience transitions in more diverse settings, we could have incorporated clerkships in primary healthcare as well. Unfortunately, neither of these different transitions could be examined due to COVID restrictions. Another limitation is the fact that we did not have permission to observe students in the presence of patients.

### Implications for future research

If we wish to gain more knowledge on how to smooth transitions across clerkships, we believe that three aspects are in need of further research. First, we need to study transitions in diverse clerkships over a longer period of time. This will provide more knowledge of the impact of transitions and the use of EPAs over the course of several clerkships, which we can use to improve our curriculum. Secondly, more knowledge of the students’ role in gaining entrustment is needed. For feedback and EPAs to be used to guide transitions even more effectively, students need to draw on feedback they received earlier and reproduce this in a new feedback request. Thirdly, more research on supervisor entrustment is needed. We would like to know more about how supervisors can be activated to entrust students on previous feedback in order to promote trust and independence for their students and take full advantage of the EPAs.

### Conclusion

This study sheds light on how an EPA-based curriculum contributes to learning processes of medical students in transitions. Our EPA-based curriculum smooths transitions by actively taking EPAs and feedback across subsequent clerkships. In this way, students build skills and confidence in order to learn and grow and work with increasing independence within the LOP. Transitions offer important learning opportunities for students, which can be more fully exploited by using EPA guidance.

## Appendix

**Appendix 1:** Master’s program of Medical Sciences.

Episode 1. Internal Medicine

Episode 2. Neurology and Psychiatry

Episode 3. Surgery

Episode 4. Pediatrics

Episode 5. Obstetrics & Gynecology and Emergency medicine

Episode 6. Otorhinolaryngology and Dermatology

Episode 7. General practice and Geriatrics

Episode 8. Senior clerkship (free of choice)
